# Clinico-Hematological Profile of Double Heterozygous Hemoglobinopathies Diagnosed by High-Performance Liquid Chromatography: A Cross-Sectional Study

**DOI:** 10.7759/cureus.111157

**Published:** 2026-06-19

**Authors:** Dipti Das, Manoj K Paswan, Anshu Jamaiyar

**Affiliations:** 1 Pathology, Rajendra Institute of Medical Sciences, Ranchi, IND; 2 Ophthalmology, Rajendra Institute of Medical Sciences, Ranchi, IND

**Keywords:** beta-thalassemia, hbe (hemoglobin e), hemoglobin s, hereditary hemoglobinopathies, high-performance liquid chromatography (hplc)

## Abstract

Background

Hemoglobinopathies are inherited disorders of hemoglobin synthesis and structure that continue to impose a significant health burden, particularly in the Indian subcontinent, where genetic diversity, endogamy, and consanguinity contribute to their high prevalence. Double heterozygous hemoglobinopathies show considerable clinical and hematological variability, and limited data are available from tribal-predominant populations of eastern India. Our study was undertaken to assess the clinico-hematological profile of double heterozygous hemoglobinopathies and to evaluate the utility of high-performance liquid chromatography (HPLC) in their accurate detection and characterization.

Methods

This study was conducted in the Department of Pathology at a tertiary care teaching hospital in eastern India over 18 months. A total of 70 patients with confirmed double heterozygous hemoglobinopathies on HPLC were included. Hematological parameters were analyzed using an automated Sysmex hematology analyzer (Sysmex Corporation, Kobe, Hyogo, Japan), and definitive diagnosis was established using cation-exchange HPLC on the Bio-Rad VARIANT II Hemoglobin Testing System (Bio-Rad Laboratories, Inc., Hercules, California, United States). Statistical analysis was performed using IBM SPSS Statistics for Windows, version 27 (IBM Corp., Armonk, New York, United States), and comparisons between groups were made using the unpaired Student’s t-test, Chi-square test, or Fisher’s exact test as appropriate.

Results

Of the 70 patients included in the study, 60 (85.7%) had Sβ-thalassemia and 10 (14.3%) had HbE-β-thalassemia. Most patients belonged to the age group of 11-20 years, with a mean age of 15.1 ± 9.9 years. Pallor and splenomegaly were the most frequent clinical manifestations. Hematological evaluation revealed moderate to severe microcytic hypochromic anemia with elevated red blood cell distribution width (RDW). Comparative analysis demonstrated significantly lower hemoglobin, packed cell volume (PCV), mean corpuscular hemoglobin (MCH), and mean corpuscular hemoglobin concentration (MCHC) values in HbE-β-thalassemia patients, whereas RDW was significantly higher (p < 0.05). HPLC analysis showed significantly elevated HbF and HbA₂ levels in HbE-β-thalassemia. No significant correlation was observed between HbF level and hemoglobin concentration (r = −0.087, p = 0.474).

Conclusion

Double heterozygous hemoglobinopathies are an important cause of chronic hemolytic anemia in eastern India, particularly among tribal populations with prevalent consanguinity. HbE-β-thalassemia showed relatively more severe anemia and anisocytosis compared with Sβ-thalassemia. HPLC proved to be a reliable tool for the accurate characterization of hemoglobin variants and compound heterozygous states. Early diagnosis, targeted screening, and genetic counseling may help reduce disease burden in high-risk populations.

## Introduction

Hemoglobinopathies are a large family of inherited hemoglobin synthesis and structure disorders, which still carry a considerable health burden worldwide and are especially problematic in low- and middle-income countries. The incidence of these disorders is very high in the Indian subcontinent due to genetic diversity, endogamy, and consanguineous marriage. A significant percentage of the population is affected by abnormal hemoglobin genes, with a wide variety of clinical phenotypes ranging from asymptomatic carrier states to transfusion-dependent anemia (TDA) [1,2]. Among these, compound or double heterozygous hemoglobinopathies represent an important clinical entity characterized by co-inheritance of two distinct abnormal globin genes. This imbalance in globin chain synthesis and abnormal hemoglobin structure causes ineffective erythropoiesis, chronic hemolysis, and a spectrum of anemias. Clinical manifestations are variable and depend on the specific genetic makeup, fetal hemoglobin levels, and other modifying factors. Signs of pallor, jaundice, splenomegaly, and growth retardation are observed, and there is considerable variation in disease severity among individuals with similar genotypes [3,4].

Hematological parameters such as hemoglobin level, red cell indices, and red cell distribution width help assess disease severity and erythropoietic activity. Double heterozygous states are often associated with microcytosis, hypochromia, and anisopoikilocytosis as a result of continuous hemolysis and ineffective erythropoiesis [5]. Diagnostic methods such as peripheral smear examination may not reliably distinguish complex hemoglobin variants and can often misidentify them, while hemoglobin electrophoresis may also be misleading. High-performance liquid chromatography (HPLC) has become an effective tool for identification and quantification of hemoglobin fractions and has proved useful in detecting abnormal hemoglobins (HbS, HbE) as well as elevated HbA₂ levels associated with β-thalassemia [6,7].

Although considerable progress has been made in understanding the clinical and hematological variability of double heterozygous hemoglobinopathies with advances in diagnostic technologies, significant differences have been observed among various populations. While some studies have reported a correlation between higher fetal hemoglobin levels and milder disease severity, others have shown inconsistent findings, suggesting the influence of additional genetic and environmental factors [8]. Furthermore, the prevalence and clinical presentation vary across regions, particularly among tribal populations, underscoring the importance of population-specific data. Limited studies from eastern India have comprehensively evaluated the clinical, hematological, and HPLC profiles of double heterozygous hemoglobinopathies. Therefore, the present study was undertaken to assess the clinico-hematological profile of double heterozygous hemoglobinopathies and to evaluate the utility of HPLC in their accurate detection and characterization.

## Materials and methods

This was a cross-sectional observational study conducted in the Department of Pathology, Rajendra Institute of Medical Sciences (RIMS), Ranchi, a tertiary care hospital and teaching institute in Jharkhand, India, over a period of 18 months (June 2024 to November 2025). Patients attending outpatient and inpatient services with clinical suspicion of hemoglobinopathy, including chronic anemia, hepatosplenomegaly, or positive sickling test, were enrolled consecutively based on predefined inclusion and exclusion criteria. The study was approved by the Institutional Ethics Committee of Rajendra Institute of Medical Sciences, Ranchi (approval number: 368, dated November 4, 2024), and all participants provided written informed consent before participation in the study.

Study population

Patients of all age groups and both sexes with confirmed double heterozygous hemoglobinopathies on HPLC and complete clinical and laboratory data were included. Patients with recent blood transfusion within the preceding three months, inconclusive HPLC findings, homozygous or single heterozygous hemoglobinopathies, and anemia due to causes other than hemoglobinopathies were excluded.

Sample size was estimated using the formula $n = \frac{Z^2 P Q}{d^2}$ at a 95% confidence interval (CI) using prevalence data from Shrivastav et al., who reported aberrant hemoglobin fractions in 7.92% of cases [9]. Using P = 7.92%, Q = 92.08%, and an allowable error of 7%, the calculated minimum sample size was approximately 58. Considering previous hospital data over the preceding two years and the exploratory nature of our study, a final sample size of 70 was considered adequate for clinico-hematological correlation analysis.

Data collection

A structured proforma with demographic data, clinical history, and examination findings was used for clinical evaluation. Hematological studies were performed with peripheral venous blood obtained by the aseptic method. CBC parameters were determined on an automated hematology analyzer (Sysmex Corporation, Kobe, Hyogo, Japan), and peripheral smear examination was done when indicated. Definitive diagnosis was made by ion exchange HPLC (Bio-Rad VARIANT II Hemoglobin Testing System using the β-thalassemia short program; Bio-Rad Laboratories, Inc., Hercules, California, United States). The amount of hemoglobin fractions (HbA₀, HbA₂, HbF, and HbS) was determined by comparing the retention time and peak shape of the hemoglobin fractions in the chromatograms obtained from the samples and standards. All samples were analyzed after internal quality control, and calibration was conducted following the manufacturer's protocol. HPLC chromatograms and hemoglobin fraction analyses were interpreted by pathologists as part of routine diagnostic evaluation.

Data analysis

The data were entered into a Microsoft Excel sheet (Microsoft Corporation, Redmond, Washington, United States) and analyzed by IBM SPSS Statistics for Windows, version 27 (IBM Corp., Armonk, New York, United States). Continuous variables were reported as mean ± standard deviation (SD), and categorical variables were reported as frequencies and percentages. The distribution of continuous variables was assessed by the Shapiro-Wilk test for assessing normality prior to analysis. Student's t-test was used appropriately for the comparisons between groups for continuous variables. The t-test was used; however, some subgroups were not equivalent, but the assumption of distributions was reasonably accepted. Pearson's correlation coefficient was used to evaluate the correlation between the levels of HbF and hemoglobin concentration. Statistical significance was defined as a p-value < 0.05.

## Results

The 70 patients studied included 60 (85.7%) with Sβ-thalassemia and 10 (14.3%) with HbE-β-thalassemia. The majority were in the age group of 11-20 years, and the average age was 15.1 ± 9.9 years. The distribution was slightly male-oriented, with the largest social group being the scheduled tribes category. Over half of the patients had consanguinity (Table 1).

**Table 1:** Demographic and baseline characteristics of study population (N = 70)

Variable	Category	Frequency (Percentage)
Age (years)	1–10	24 (34.3%)
	11–20	25 (35.7%)
	21–30	16 (22.9%)
	>30	5 (7.1%)
Sex	Male	39 (55.7%)
	Female	31 (44.3%)
Social Stratification	Scheduled Tribe	27 (38.6%)
	Muslim	18 (25.7%)
	Scheduled Caste	15 (21.4%)
	Others	10 (14.3%)
Consanguinity	Present	41 (58.6%)
	Absent	29 (41.4%)

**Figure 1:**
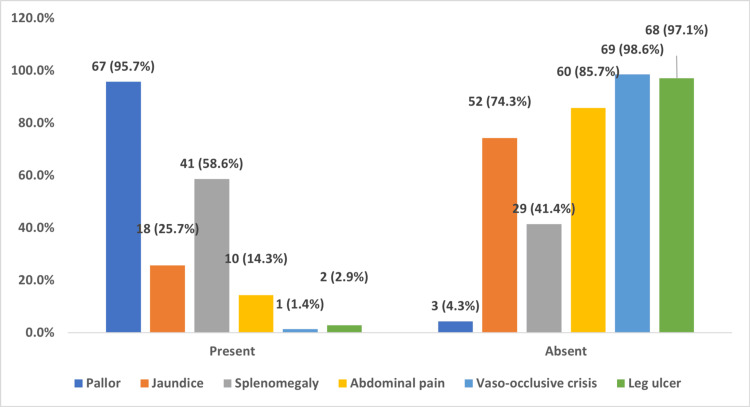
Distribution of clinical features in double heterozygous hemoglobinopathies

Pallor was the most common clinical sign, followed by splenomegaly, jaundice, and rare occurrence of vasocclusive crises and leg ulcers (Figure 1).

Moderate to severe microcytic hypochromic anemia with elevated red blood cell distribution width (RDW) (significant anisocytosis) was noted on hematological evaluation (Table 2).

**Table 2:** Hematological parameters of study population (N=70) RBC: red blood cell count; PCV: packed cell volume; MCV: mean corpuscular volume; MCH: mean corpuscular hemoglobin; MCHC: mean corpuscular hemoglobin concentration; RDW: red blood cell distribution width

Parameter	Mean ± SD	Reference Range
Hemoglobin (g/dL)	7.37 ± 2.51	1.8-14.4
RBC (×10⁶/µL)	3.07 ± 0.98	0.89-4.87
PCV (%)	24.09 ± 7.65	6.4-44.6
MCV (fL)	70.70 ± 8.53	50.2-100.2
MCH (pg)	24.11 ± 4.24	15.1-32.5
MCHC (g/dL)	30.13 ± 3.11	23.3-38.4
RDW (%)	22.89 ± 5.38	12.8-38.1
Reticulocyte (%)	2.34 ± 1.13	1.0-5.74

Our analysis showed significant differences between the HbE-β-thalassemia patients and the Sβ-thalassemic patients in terms of hemoglobin, PCV, MCH, and MCHC. The RDW was significantly higher in the HbE-β-thalassemic group, suggesting more anisocytosis and disease severity (Table 3).

**Table 3:** Comparison of hematological parameters between groups *Statistically significant (p < 0.05) RBC: red blood cell count; PCV: packed cell volume; MCV: mean corpuscular volume; MCH: mean corpuscular hemoglobin; MCHC: mean corpuscular hemoglobin concentration; RDW: red blood cell distribution width; S-β: sickle beta; HbE-β: hemoglobin E-beta

Parameter	Sβ-Thalassemia (n=60)	HbE-β-Thalassemia (n=10)	t-test	p-value
Hemoglobin (g/dL)	7.66 ± 2.49	5.58 ± 1.88	3.08	0.014*
RBC (×10⁶/µL)	3.12 ± 0.99	2.75 ± 0.85	1.24	0.265
PCV (%)	24.85 ± 7.54	19.51 ± 7.03	2.20	0.040*
MCV (fL)	70.91 ± 8.04	69.46 ± 11.54	0.38	0.632
MCH (pg)	24.75 ± 3.90	20.25 ± 4.32	3.09	0.001*
MCHC (g/dL)	30.54 ± 2.86	27.69 ± 3.61	2.38	0.006*
RDW (%)	22.09 ± 4.32	27.68 ± 8.34	2.07	0.002*

HPLC analysis demonstrated distinct hemoglobin fraction patterns between the two groups. HbE-β-thalassemia showed significantly higher HbF and HbA₂ levels, whereas HbS was characteristically detected in Sβ-thalassemia (Table 4).

**Table 4:** HPLC profile and hemoglobin fraction comparison *Statistically significant (p < 0.05) HbF: fetal hemoglobin; HbA₀: adult hemoglobin A₀; HbA₂: hemoglobin A₂; HPLC: high-performance liquid chromatography; S-β: sickle beta; HbE-β: hemoglobin E-beta

Parameter	Overall Mean ± SD	Sβ-Thalassemia	HbE-β-Thalassemia	t-test	p-value
HbF (%)	21.93 ± 6.83	21.03 ± 6.25	27.34 ± 7.92	2.82	0.006*
HbA₀ (%)	9.12 ± 10.44	8.91 ± 10.87	10.40 ± 7.71	0.42	0.678
HbA₂ (%)	11.74 ± 18.24	4.54 ± 1.05	54.95 ± 11.06	14.31	<0.001*

HbS fraction was detected in Sβ-thalassemia and was not identified in HbE-β-thalassemia cases. Correlation analysis using Pearson’s correlation coefficient showed no significant association between HbF levels and hemoglobin concentration (r = −0.087, p = 0.474), suggesting that HbF alone may not reliably predict disease severity in double heterozygous hemoglobinopathies.

## Discussion

Our study evaluated the clinico-hematological and chromatographic profile of double heterozygous hemoglobinopathies and demonstrated that these disorders predominantly affect children and young adults with variable clinical severity and distinct HPLC patterns. Similar early presentations have been reported by Balgir [10] and Mohanty et al. [11], who attributed this to chronic hemolysis and ineffective erythropoiesis in Indian populations. A slight male predominance observed in our study was comparable to findings by Nayar et al. [12]. A substantial proportion of patients belonged to tribal communities, and consanguinity was frequently observed. Verma et al. [13] and Balgir [10] highlighted the contribution of endogamy and consanguineous marriages to the persistence of abnormal hemoglobin genes in tribal populations. These findings highlight the importance of screening and genetic counseling in high-risk areas.

Pallor and splenomegaly were the most frequent clinical manifestations, similar to findings reported by Lakhani et al. [14] and Jain et al. [15]. The lower frequency of vaso-occlusive crises in this group of patients may be attributed to relatively higher levels of HbF, which are known to decrease the severity of sickling [3]. Hematological analysis revealed moderate-to-severe microcytic hypochromic anemia and elevated RDW, which was more pronounced in HbE-β-thalassemia patients compared to Sβ-thalassemia patients, similar to observations reported by Shrivastav et al. [9] and Mansoor et al. [16]. Comparative analysis showed that HbE-β-thalassemia patients had more severe anemia and greater anisocytosis than Sβ-thalassemia patients. Similar findings were reported by Fucharoen and Weatherall, who described HbE-β-thalassemia as a clinically heterogeneous disorder with variable but often severe manifestations [17]. The greater severity observed in HbE-β-thalassemia may be related to ineffective erythropoiesis, variable β-globin suppression, and additional nutritional or environmental modifiers.

HPLC analysis demonstrated characteristic hemoglobin fraction patterns, supporting its utility in the accurate identification of compound hemoglobinopathies. Joutovsky et al. emphasized the diagnostic value of HPLC in differentiating hemoglobin variants based on retention times and quantitative hemoglobin fractions [6]. Although elevated HbF levels are generally considered protective, no significant correlation was observed between HbF level and hemoglobin concentration in our study, suggesting that additional genetic and environmental factors may influence disease severity [8].

Strengths and limitations

The strengths of our study include comprehensive clinico-hematological evaluation with HPLC-based characterization and the provision of region-specific data from a tribal-predominant population. However, the relatively small sample size, single-center design, and lack of molecular analysis may limit broader generalizability and detailed genotype-phenotype correlation. Also, some of these comparisons are performed in subgroups that are of unequal size, which might have introduced statistical bias in the analyses, even though the analyses were conducted using standard parametric methods. Another limitation of the present study is that HPLC results were obtained but not molecularly confirmed routinely. Though it is a fast, reliable, and widely used screening test for common hemoglobin variants and thalassemias, HPLC is not effective in distinguishing complex hemoglobinopathies, co-inherited disorders, or rare variants that might have similar retention time. However, HPLC is not sufficient to identify certain conditions, e.g., compound heterozygous states, silent β-thalassemia mutations, and rare structural hemoglobin variants. In these situations, molecular diagnostic methods such as polymerase chain reaction (PCR)-based assays and DNA sequencing continue to be the gold standard for definitive diagnosis. Some complex hemoglobinopathy patterns were therefore not confirmed by molecular studies in our study, which could have resulted in misclassification of some patterns and should be taken into consideration when interpreting the results. And also, as this was a retrospective study based on existing laboratory records, blinding of investigators to patient information was not performed.

## Conclusions

Double heterozygous hemoglobinopathies are an important cause of chronic hemolytic anemia in eastern India, particularly among tribal populations with prevalent consanguinity. HbE-β-thalassemia showed relatively more severe anemia and anisocytosis compared with Sβ-thalassemia. HPLC proved to be a reliable tool for accurate characterization of hemoglobin variants and compound heterozygous states. Early diagnosis, targeted screening, and genetic counseling may help reduce disease burden in high-risk populations. Larger multicentric studies with molecular correlation are warranted.
